# The Study of the Distribution of Electrical and Magnetic Properties over the Conductor Cross-Section Using Magnetoimpedance Tomography: Modeling and Experiment

**DOI:** 10.3390/s22239512

**Published:** 2022-12-05

**Authors:** Dmitry A. Bukreev, Michael S. Derevyanko, Alexey A. Moiseev, Andrey V. Svalov, Alexander V. Semirov

**Affiliations:** 1 Department of Physics, Pedagogical Institute, Irkutsk State University, 664003 Irkutsk, Russia; 2 Department of Magnetism and Magnetic Nanomaterials, Institute of Natural Sciences and Mathematics, Ural Federal University, 620002 Ekaterinburg, Russia

**Keywords:** magnetoimpedance effect, amorphous magnetically soft alloys, computer simulation, finite element method

## Abstract

A description of the method of magnetoimpedance tomography is presented. This method is based on the analysis of the frequency dependences of the impedance obtained in magnetic fields of various strengths. It allows one to determine the distribution of electrical and magnetic properties over the cross-section of the conductor, as well as their dependence on the magnetic field. The article proposes a specific approach to the implementation of the magnetoimpedance tomography method based on computer modeling by the finite element method. The results of this method are presented for composite Cu_98_Be_2_/Fe_20_Co_6_Ni_74_ wires of the “highly conductive core–magnetically soft coating” type and amorphous rapidly quenched Co_66_Fe_4_Nb_2.5_Si_12.5_B_15_ wires.

## 1. Introduction

The dependence of the complex electrical resistance of a ferromagnetic conductor on an external magnetic field’s strength is called the magnetoimpedance effect (hereinafter—MI) [1]. MI offers wide opportunities for the development of compact devices for small magnetic field detection up to the level of biomagnetic fields [2,3]. If we confine ourselves to objects of cylindrical geometry, then MI is actively studied in amorphous cobalt-based wires obtained by rapid quenching from a melt [1,4], microwires in a glass shell [5], and composite wires consisting of a highly conductive core–magnetically soft coating [6,7], obtained by electrolytic deposition. The MI in these objects can reach hundreds of percentages, even in relatively weak magnetic fields, which allows them to be used in highly sensitive magnetic field sensors [8,9,10].

The MI can be clearly explained in terms of the classic electrodynamics skin effect. When an alternating current flows through the ferromagnetic conductor, the effective cross-section of it can be determined by the thickness of the skin layer, as follows [11]:

(1) $$\delta =\sqrt{\frac{1}{\pi f\sigma \mu_{0}\mu }},$$

where *f* is the frequency of the alternating current (AC), *µ*_0_ is the magnetic constant, *µ* is the effective transverse (relative to the direction of the AC) magnetic permeability, and *σ* is the specific conductivity. A change in the magnetic field leads to a change in the magnetic permeability. This, in turn, leads to a change in *δ*. The more *δ* differs from the radius of the cylindrical conductor, the more the impedance modulus differs from the DC resistance *R*_DC_. If we neglect the contribution of the external part of the self-induction, then we can obtain the following expression by solving the Maxwell equations for the impedance of a homogeneous cylindrical conductor of radius *r* [11]:

(2) $$\frac{\dot{Z}}{R_{\mathrm{DC}}}=\frac{1}{2}kr\frac{J_{0}\left(kr\right)}{J_{1}\left(kr\right)},$$

where *R*_DC_ is the resistance of the conductor to direct current, *k* = (1 + *i*)/*δ*; *i* is the imaginary unit; and *J*_0_ and *J*_1_ are the Bessel functions of the first kind of zero and first orders.

At the same time, both the amorphous and the composite wires are characterized by a non-uniform radial distribution of electromagnetic parameters. The magnetic structure of the amorphous, magnetically soft wire obtained by rapid quenching from melt [12] can be simplified by the core-shell model. According to this model, the wire has an axially magnetized core and a radially or circularly magnetized shell [13]. This distribution of the magnetic properties is determined by quenching stresses.

The composite wire of the highly conductive core–thin magnetically soft coating type contains at least two axial regions with different electrical conductivities and magnetic permeabilities. The size and composition of these areas are set during the manufacturing of the composite wire [14,15]. The solution of Maxwell’s equations does not lead to expressions as simple as Expression (2) due to the complex radial distribution of electromagnetic properties over the cross-section of amorphous and composite wires.

For the case of amorphous wires, in particular, the MI was described in the case of the presence of two regions—the central, axially magnetized (core), and the near-surface, circularly magnetized (shell) [16]. In addition, the influence of the domain wall between the core and shell was considered [17]. Moreover, a theoretical study of the influence of the radial distribution of magnetic anisotropy axes on the MI was carried out [18].

A rigorous theoretical description of the MI of the composite wires was discussed in [19,20]. In some cases, the calculation can be simplified. For example, the impedance can be represented as the sum of contributions from the weakly magnetic core and the coating of the composite wire if the coating is sufficiently thin, and its electrical conductivity is much less than the specific electrical conductivity of the core [21].

It is important to note that the thickness of the skin layer depends not only on the electromagnetic parameters of the conductor but also on the frequency of the alternating current. The expression (1) shows that the higher f, the smaller *δ*. In other words, as the AC frequency increases, the contribution to the impedance from the inner regions decreases, while the contribution from the surface regions, on the contrary, increases. Due to this, it is possible to restore the distribution of the electromagnetic parameters over the conductor cross-section using the dependences of the MI on the AC frequency. This method can be called magnetoimpedance tomography.

Magnetoimpedance tomography may be in demand for determining the optimal modes of operation of amorphous and composite wires. Thus, in the case of composite wire, according to some experimental and theoretical results, it is possible to control the optimum operating modes by varying the geometric dimensions of the core and coating and their electromagnetic properties, including the features of effective magnetic anisotropy [19,22,23]. The MI response of an amorphous wire can be changed by varying its composition [24,25,26] or by different heat treatments [27,28]. It is possible to purposefully adjust the characteristics of the MI-sensitive elements to specific tasks if the radial distributions of electromagnetic parameters are known. This can be achieved by the appropriate selection of manufacturing conditions and further heat treatment.

Note that attempts have already been made to restore the radial distribution of the magnetic permeability of amorphous microwires using Expression (2) [29]. However, the use of this method is valid only with serious reservations due to the inhomogeneous radial distribution of the electromagnetic properties. At the same time, the problem of magnetoimpedance tomography based on the expressions obtained in [16,17,18] and [19,20] seems to be difficult to solve.

Here, it may be useful to solve Maxwell’s equations with the help of computer modeling using the finite element method. This approach has proven itself well in solving problems related to MI [30,31]. It will be possible to find an approximation of the real distribution of magnetic permeability by modeling the frequency dependences of the impedance in cylindrical conductors at different radial distributions of magnetic permeability and specific conductivity and comparing the modeled dependences with the experimental ones. In future investigations, it will be possible to take into account that the cross-section of the wires, in reality, is not strictly circular, and also to study planar MI elements, such as amorphous ribbons and multilayer films, which, in particular, can be used in the tasks of a bio-detection [32,33].

This work presents the results of the computer simulation of the impedance response of the cylindrical conductor at different radial distributions of the magnetic permeability and specific conductivity. The simulation results are compared with the experimental dependences of the impedance on AC frequency obtained in the study of the composite wire CuBe/FeCoNi and rapidly quenched CoFeNbSiB amorphous wire in magnetic fields of different strengths. As a result of the comparison, the radial distributions of the specific conductivity and the magnetic permeability are determined, which is the basis of the magnetic impedance tomography method.

## 2. Materials and Methods

The following cylindrical conductors were investigated experimentally:

- The composite wires were obtained by the electrolytic deposition of Fe_20_Co_6_Ni_74_ ferromagnetic alloy (coating) on Cu_98_Be_2_ wire (core). The thickness of the deposited layer was 1 µm with a total wire radius of 52.5 µm. The coating thickness was determined by both deposition time and scanning electron microscopy performed on Hitachi TM3000. Magnetoimpedance measurements were performed on the 30 mm-long samples. Here, it should be noted that the electrolytic technology for obtaining the magnetic layer causes the formation of circular magnetic anisotropy in the initial samples [18]. In this case, the specific parameters of the magnetic anisotropy of the samples can have a certain dispersion. Thus, the initial wires were subjected to thermomagnetic treatment for 1 h at a temperature of 600 K in the axial magnetic field with a strength of 12 kA/m to induce the axial magnetic anisotropy with the lowest possible dispersion.
- Amorphous Co_66_Fe_4_Nb_2.5_Si_12.5_B_15_ wires with a radius of 90 µm were prepared by the in-rotating-water quenching technique [12]. The structural state of these wires was discussed by us earlier [28]. Moreover, the studies of other authors indicate the amorphous state of wires similar in composition and geometry [34].

The saturation magnetization, *M_s_*, of the wires at room temperature was about 320 kA/m. Samples 100 mm long were used for the magnetoimpedance measurements. The length of the samples was chosen from the considerations of minimizing the effect of closure domains on the distribution of the magnetization over the cross-section. The samples were not heat-treated.

The experimental frequency dependences *Z*(*f*)/*R*_DC_ were obtained using an automated complex of magnetoimpedance spectroscopy, which was developed by the authors and described in the work [35]. The alternating current flowed through a section of the sample 24 mm long and was located in its middle. The effective value of the current strength was 1 mA, and the frequency varied in a range from 0.01 to 80 MHz. The external magnetic field, *H*, was oriented along the length of the sample. The maximum value of its strength, *H_max_*, was 12 kA/m.

The MI ratio was calculated using the formula:

(3) $$\Delta Z/Z\left(H\right)=\frac{Z\left(H\right)-Z\left(H_{max}\right)}{Z\left(H_{max}\right)}\;\cdot \;100\%,$$

where *Z*(*H*) and *Z*(*H_max_*) are the wire impedance moduli measured in magnetic fields *H* and *H_max_*, respectively.

The magnetic hysteresis loops were obtained by the induction method. The magnetic field varied with a frequency of 1 kHz and their amplitude was 2 kA/m.

The cross-sectional analysis of the elements by energy-dispersive X-ray spectroscopy (EDX) was performed using an EDX detector of the scanning electron TM3000 microscope (HITACHI Ltd., Tokyo, Japan).

### 2.1. Computer Simulation

**Figure 1:**
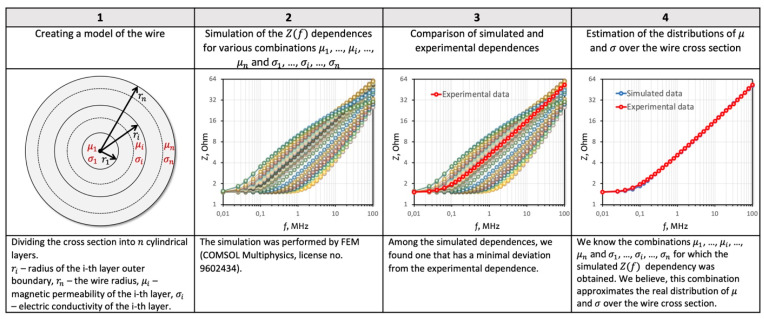
Algorithm for comparing the simulated and experimental frequency dependences of the impedance.

The *Z*(*f*)/*R_DC_* dependences were modeled by the finite element method using the Comsol Multiphysics software package (license No. 9602434) in the *f* range from 0.01 to 80 MHz. The wire model consisted of n coaxial cylindrical regions with outer boundaries of radii *r_i_* (Figure 1).

1. When modeling the composite wire, *n* was equal to 2. In this case, *r_1_* was equal to 51.5 µm, which corresponded to the core radius. The value of *r_2_* was equal to the wire radius, 52.5 µm. Thus, the electrodeposited coating was modeled by the area enclosed between *r*_1_ and *r*_2_. The electrical conductivity of the core, *σ*_1_, varied from 15 to 20 MS/m with a step of 0.2 MS/m, and its magnetic permeability was taken as 1. The specific electrical conductivity of the coating, *σ*_2_, varied in the range from 0.5 to 4 MS/m with a step of 0.2 MS/m, and the magnetic permeability of this region, *µ*_2_, varied from 1 to 1500 with a step of 1. All these values varied independently. In this way, an array of the dependences of the reduced impedance on the frequency of the alternating current *Z*(*f*)/*R_DC_* was obtained, corresponding to various combinations of values of *σ*_1_, *σ*_2_, and *µ*_2_.
2. When modeling amorphous CoFeNbSiB wire, the cases with *n* equal to 2 and 3 were considered (see Table 1). The total radius of the amorphous wire model was *r_n_* = 90 µm. The value of the *r*_1_ varied from 70 to 85 m. For *n* = 3, three variants with *r*_2_ equal to 87, 88, and 89 µm were considered. The electrical conductivity of all regions, *σ*, was taken equal to 0.87 MS/m. The magnetic permeability of each region *µ_i_*, regardless of the permeabilities of other regions, varied from 1 to 15,000 with a step of 10 to 50. As a result, an array of the *Z*(*f*)/*R_DC_* dependences corresponding to the various combinations of magnetic permeabilities *µ_i_* was obtained.

The frequency dispersion of the magnetic permeability and electrical conductivity, as well as their complex and tensor character, was not taken into account. When constructing a finite element mesh, it was ensured that the size of its element did not exceed the thickness of the skin layer [30].

### 2.2. Implementation of the Method of the Magnetoimpedance Tomography

In the array of the simulated *Z*(*f*)/*R_DC_* dependences, a dependence was found that had the smallest absolute deviation from the experimental dependence (Figure 1). As a result, a combination of the magnetic permeability and electrical conductivity of the wire model regions was obtained, which, presumably, is an approximation of the real radial distribution of these values.

## 3. Results and Discussion

### 3.1. Composite Wires

**Figure 2:**
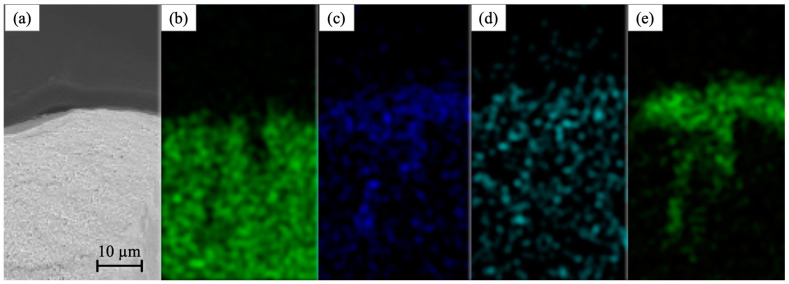
The composite wire images obtained by scanning electron microscopy (**a**) and X-ray fluorescence at wavelengths corresponding to Cu-Kα (**b**), Fe-Kα (**c**), Co-Kα (**d**), and Ni-Kα (**e**).

An X-ray fluorescence study showed that the core, as expected, mainly gives the characteristic radiation of Cu-K_α_ (Figure 2). The characteristic radiations of the coating material in descending order of intensity are Ni-K_α_, Fe-K_α_, and Co-K_α_. These results are expected considering the composition of the Fe_20_Co_6_Ni_74_ alloy used for the electrodeposition.

**Figure 3:**
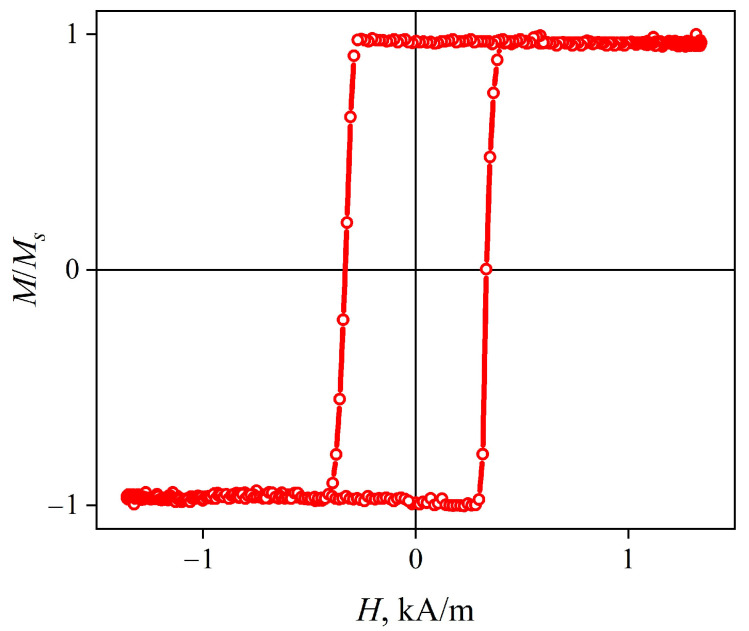
Longitudinal (measured along the wire axis) magnetic hysteresis loop of the composite wire CuBe/FeCoNi.

The longitudinal magnetic hysteresis loop of the CuBe/FeCoNi composite wire has convexity and squareness coefficients close to 1 (Figure 3). Such a loop indicates that axial magnetic anisotropy with a very small dispersion was formed during the annealing. The coercive field is approximately 0.32 kA/m.

**Figure 4:**
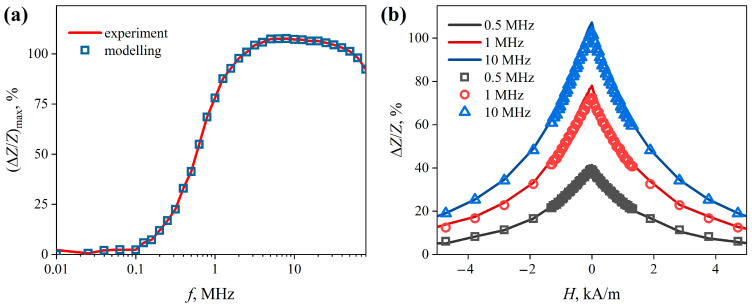
Dependence of the maximum MI value of the CuBe/FeCoNi composite wire on the AC frequency (Δ*Z*/*Z*)*_max_*(*f*) (**a**). Dependences of the MI of the CuBe/FeCoNi composite wire on the strength of the external magnetic field Δ*Z*/*Z*(*H*), obtained at AC frequencies of 0.5, 1, and 10 MHz (**b**). The lines correspond to the experimental dependences, the markers correspond to the values obtained as a result of modeling by the finite element method.

At AC frequencies up to 0.1 MHz, the magnetoimpedance effect is weakly expressed (Figure 4a). As can be seen from the figure, with a further increase in the frequency, the maximum MI value, (Δ*Z*/*Z*)*_max_*, rapidly increases, reaching values of more than 100% near 4 MHz, and then demonstrates a tendency to decrease. Dependences of the MI on the strength of the external magnetic field, Δ*Z*/*Z*(*H*), at AC frequencies above 0.1 MHz, have the form of a “single peak” (Figure 4b) [36]. In other words, the maximum value of the magnetoimpedance effect (Δ*Z*/*Z*)*_max_* is observed at *H* = 0 (Figure 4b, solid lines).

**Figure 5:**
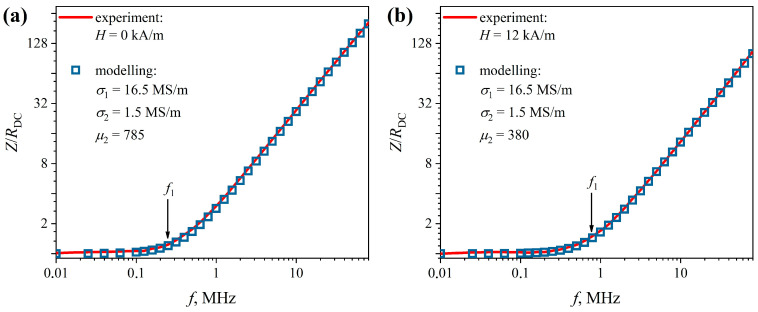
Dependences of the reduced impedance *Z*/*R_DC_* of the CuBe/FeCoNi composite wire on the AC frequency, *f*, obtained at the external magnetic field strength 0 (**a**) and 12 kA/m (**b**).

The experimental frequency dependences of the reduced impedance *Z*(*f*)/*R_DC_* of the composite wire are shown in Figure 5 (solid lines). On a logarithmic scale, the dependence *Z*(*f*)/*R_DC_* can be described by two linear segments—almost horizontal and increasing—for all studied external magnetic field strengths. The first section smoothly passes into the second one at a certain frequency of the alternating current, which we denote by *f_1_*. For example, at *H* = 0 it is approximately 0.4 MHz. With an increase in the external magnetic field strength, *f_1_* increases, which is caused by a change in the magnetic permeability of the coating.

It is clear from Expressions (1) and (2) that the horizontal section of the *Z*(*f*)/*R_DC_* dependence corresponds to the situation when the skin layer thickness *δ* is greater than the wire radius. Therefore, for *f* < *f*_1_, the impedance modulus *Z* ≈ *R_DC_*. For *f* > *f*_1_, the skin layer thickness decreases with increasing frequency, leading to an increase in the impedance. It should be noted that near the frequency *f*_1_, the current is mainly distributed along the core. However, the magnetic permeability of the coating strongly affects this distribution, which is reflected in the change in this characteristic frequency when the external magnetic field strength changes.

Then, using magnetoimpedance tomography (see Section 2.2), the distribution of electromagnetic parameters over the composite wire section can be determined. We determined that the specific conductivity of the core is *σ*_1_ = 16.5 MS/m, and the conductivity of the coating is *σ*_2_ = 1.5 MS/m. The first value of the conductivity is typical for CuBe alloys from which the core is made [37], and the second value falls within the range of conductivity values characteristic of permalloys. In this case, the calculation according to the formula

(4) $$R_{\mathrm{DC}}=\frac{l}{\pi \left(r_{1}^{2}\sigma_{1}+\left(r_{2}^{2}-r_{1}^{2}\right)\sigma_{2}\right)}$$

gives the value of the electrical resistance, which practically coincides with the experimentally measured one.

The magnetic permeability of the coating was different for each value of the external magnetic field strength *H*. For example, for *H* = 0, we obtained *µ_2_* = 785 (Figure 5a), and for *H* = 12 kA/m, we obtained *µ*_2_ = 380 (Figure 5b). Note that the relative deviation of the simulated dependences *Z*(*f*)/*R_DC_* from the experimental ones did not exceed 3% for the entire studied AC frequency and the magnetic field ranges.

**Figure 6:**
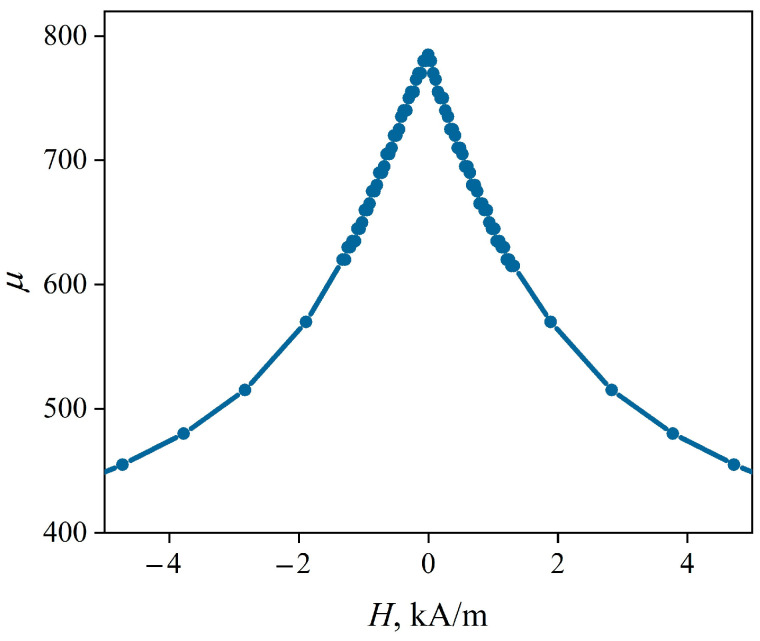
Magnetic field dependence of the magnetic permeability of the CuBe/FeCoNi composite wire coating, reconstructed using magnetoimpedance tomography.

The field dependence of the magnetic permeability *µ*_2_(*H*) reconstructed using magnetoimpedance tomography is shown in Figure 6. It can be seen that *µ*_2_(*H*) reaches a maximum value of 785 in the zero external magnetic field, indicating a predominantly longitudinal orientation of the magnetic anisotropy axis. The same conclusion was obtained above from the analysis of the hysteresis loop (Figure 3).

Using the dependence *µ*_2_(*H*), as well as the values of *σ_1_* and *σ_2_*, obtained using the magnetoimpedance tomography, we reconstructed the magnetoimpedance dependences Δ*Z/Z*(*H*) (Figure 4b, empty markers). It is clearly seen that the simulated dependences practically coincide with the experimental ones. The frequency dependences of the maximum MI value are also in good agreement (Figure 4a, empty markers).

A more detailed study of the radial distribution of the electromagnetic parameters over the wire cross-section is possible with an increase in the number of layers *n* during simulation. In particular, this may be required in the study of the mutual diffusion of materials of the core and coating, as a result of which their electrical and magnetic properties change [38].

### 3.2. Amorphous CoFeNbSiB Wires

In the case of the amorphous wires, the boundaries of the regions are not known in advance, as in the case of the composite wires described above. This leads to the need to consider models with different values of *r_i_*. On the other hand, it can be assumed that the electrical conductivity σ is the same at all points of the wire cross-section, which simplifies the task.

**Figure 7:**
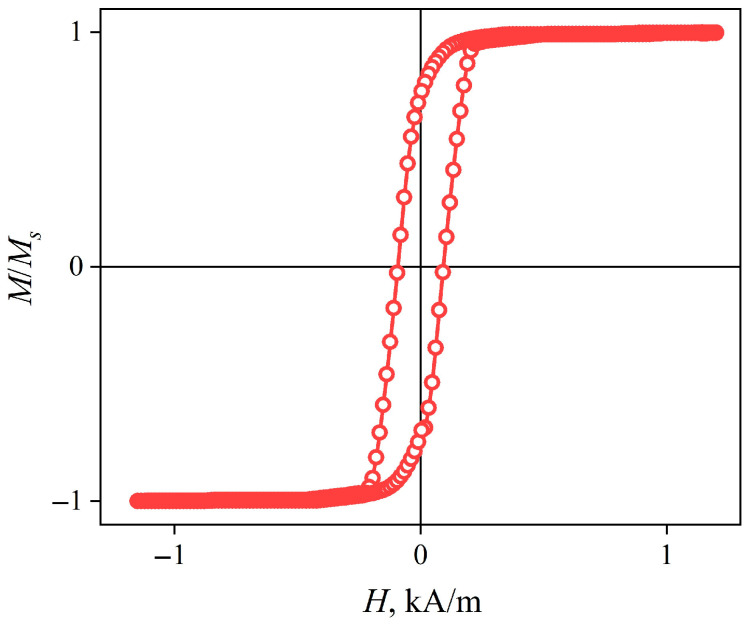
Hysteresis loop of an amorphous wire Co_66_Fe_4_Nb_2.5_Si_12.5_B_15_.

**Figure 8:**
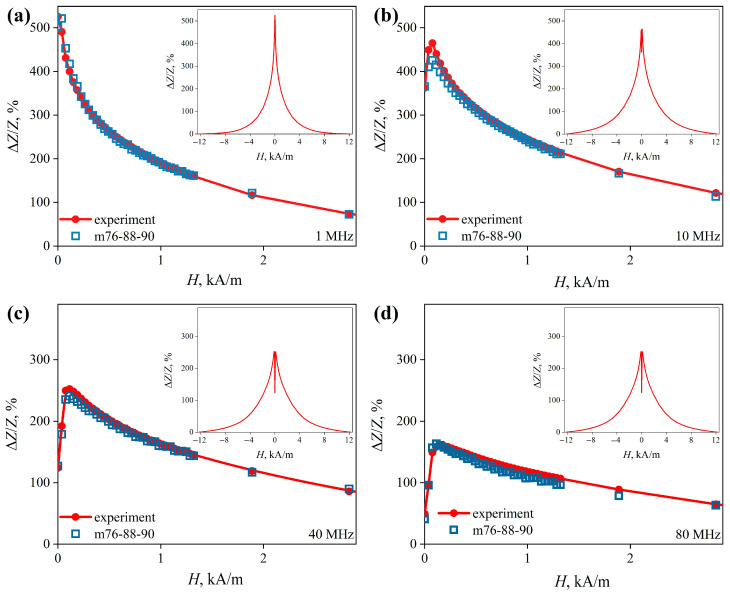
MI behavior for an amorphous Co_66_Fe_4_Nb_2.5_Si_12.5_B_15_ wire. The line with round markers shows the dependences of the magnetoimpedance effect on the strength of the external magnetic field Δ*Z/Z*(*H*), obtained at AC frequencies: (**a**) 1 MHz, (**b**) 10 MHz, (**c**) 40 MHz, and (**d**) 80 MHz. The insets show the same dependences in the range of the magnetic fields from –12 to 12 kA/m. Square markers are data obtained using the m76-87-90 model.

The hysteresis loop shows that the magnetic anisotropy of the wire is predominantly axial (Figure 7). More detailed information about the radial distribution of the magnetic anisotropy can be obtained from the dependences of the MI on the external magnetic field strength Δ*Z/Z*(*H*) (Figure 8).

When *f* < 2.5 MHz, the Δ*Z/Z*(*H*) dependences have the form of a “single peak”, and at higher frequencies, they have the form of a “double peak” [35] (Figure 8a–d, insets). A change in the type of dependences with an increase in the frequency of the alternating current indicates the presence in the wire of at least two regions with different orientations of magnetic anisotropy axes. At low AC frequencies, the skin layer thickness is large, so the MI response is formed mainly due to the central region of the wire, which is predominantly axially magnetized, and we observe the “single peak” dependence. When the frequency increases, the thickness of the skin layer decreases, so the contribution of the central region of the wire also decreases, while the contribution of the outer region increases. Thus, the Δ*Z/Z*(*H*) dependences change to the “two peaks” form since the magnetic anisotropy of the outer region apparently has a significant circular component. For the same reason, the magnetic field intensity *H_p_*, corresponding to the maximum in the Δ*Z/Z*(*H*) dependence, increases with the increase in the AC frequency. At the upper limit of the studied frequency range, *H_p_* is about 0.15 kA/m.

**Figure 9:**
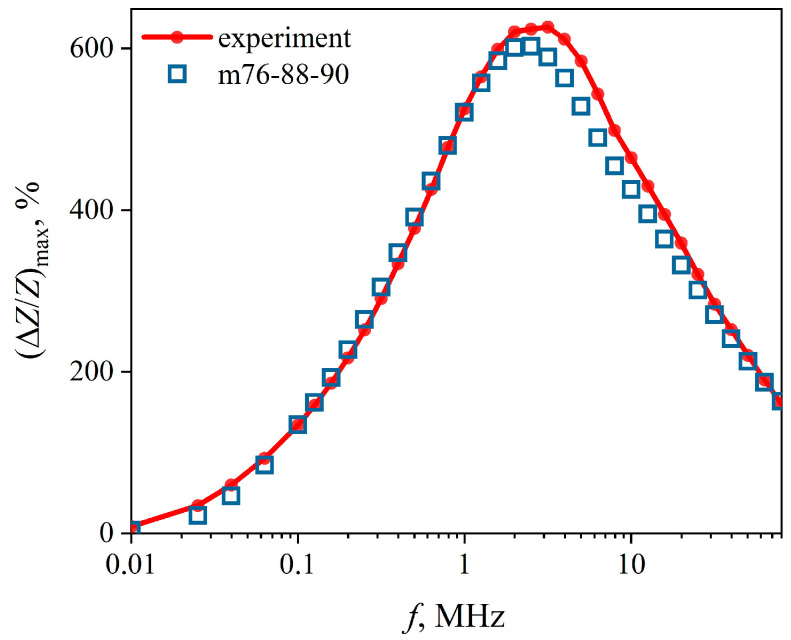
Dependences of the maximum value of the magnetoimpedance effect on the AC frequency (Δ*Z*/*Z*)max(*f*), calculated on the basis of data obtained both experimentally and using the m76-87-90 model.

Note that the MI reaches a very high value close to 600% near the above-mentioned AC frequency of 2.5 MHz. (Figure 9).

It should be noted that the response of the “double peak” type is of interest for practical application, since in this case, in the field range from 0 to ± *H_p_*, a very high sensitivity is achieved, exceeding that in the case of the MI response of the “single peak” type [39]. Therefore, MI elements with such a response are used for the tasks of detecting ultra-low magnetic fields in bioengineering and biomedical applications [40,41]. The improvement and/or formation of the “double peak” response in the case of amorphous wires is possible, for example, by annealing with an electric current [28,42].

**Figure 10:**
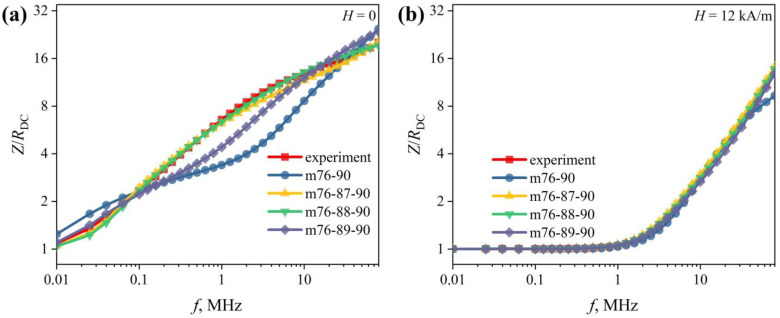
Reduced impedance versus AC frequency *Z*(*f*)/*R_DC_*. Experimental results were obtained in the magnetic fields *H* with intensity (**a**) 0; (**b**) 12 kA/m. The values of the magnetic permeabilities *μ_i_* used in the modeling are shown in the figure.

Next, a comparison between the experimental frequency dependences of the impedance *Z*(*f*)/*R_DC_* and the simulation results was made (Figure 10). For *n* = 2, the best result was obtained using the m76-90 model (see Table 1). However, even this did not allow for achieving a satisfactory agreement between the calculated data and the experimental data. In the field *H* ≤ *H_p_*, the absolute deviation of the modeled dependence from the experimental one exceeds 50% (Figure 10). At *H* > *H_p_*, the deviation decreases, but still exceeds 20%. The reason for such a large deviation is probably that the simple two-layer model gives a too-rough approximation of the real radial distribution of the magnetic permeability in the wire.

Note that the radial distribution of the magnetic permeability near the wire surface can be significantly affected by roughness on its surface [43,44]. Based on this, we introduced a third layer into the model, and *r_2_* (the radius of the inner boundary of the third layer) varied from 87 to 89 µm (see Table 1). The introduction of the third layer noticeably brings the simulated and experimental dependences closer together (Figure 10). The m76-87-90 model gives the best result. However, at AC frequencies below 0.1 MHz and *H* ≤ *H_p_*, the deviation of the simulated dependence from the experimental one reaches 10%. In all other cases, it does not exceed 5%. Interestingly, for *H* > *H_p_*, all three-layer models give similar results. Perhaps this is due to the fact that as the wire approaches saturation, the radial distribution of the magnetic permeability becomes more uniform than in the fields *H* ≤ *H_p_*.

**Figure 11:**
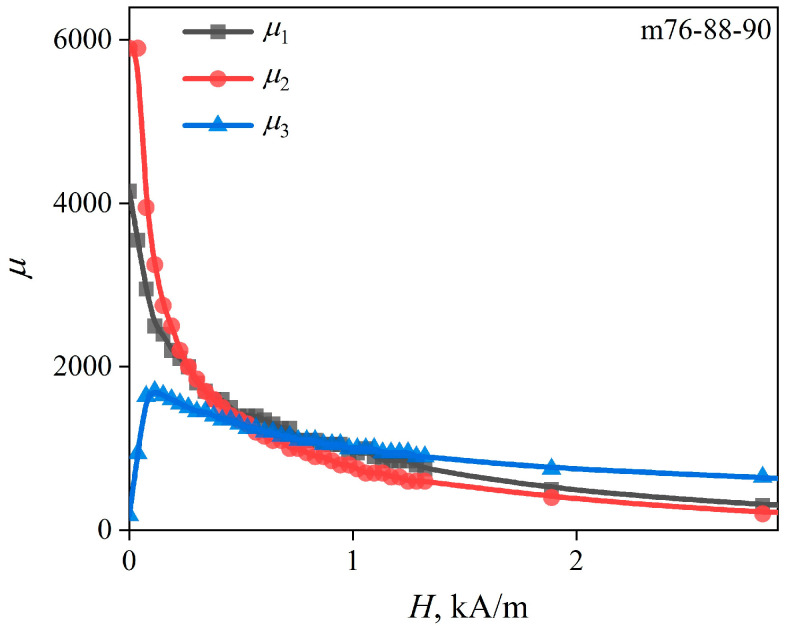
The values of the magnetic permeability of the layers *µ_i_* of the m76-87-90 model, corresponding to different intensities of the external magnetic field *H*.

The dependences of the magnetic permeability of the wire regions (*r_1_*, *r_2_*, and *r_3_*) on the external magnetic field strength *µ_i_*(*H*) were reconstructed (Figure 11) after comparing *Z*(*f*)/*R_DC_* dependences, calculated using the m76-87-90 model with the experimental ones measured in magnetic fields with strengths from 0 to *H_max_*, as described in Section 2.2.

The magnetic permeability of the central layer *µ_1_* has a maximum in the zero external magnetic field, as well as the permeability *µ_2_*. In this case, *µ_2_* is noticeably larger than *µ*_1_. As *H* increases, the field dependences of the permeabilities converge.

The field dependence of the magnetic permeability of the outer layer *µ_3_*(*H*) has a maximum field strength of 0.15 kA/m. Note that this field is close to the previously mentioned *H_p_* field. In the zero field, the value of *µ_3_* is about unity, which indicates a predominantly circular anisotropy of the near-surface wire layer.

The Δ*Z/Z*(*H*) dependences were calculated (Figure 8, square markers) using the *µ_i_*(*H*) dependences (Figure 11). We found that they are very close to the experimental ones. The same can be said about the frequency dependence of the maximum value of the MI (Figure 9).

The absence of the increasing section in the *µ_1_*(*H*) and *µ_2_*(*H*) dependences does not indicate a strictly axial anisotropy. To show this, let us turn to the expression for the magnetic permeability obtained from the model of the uniform rotation of the magnetization, as described, for example, in [35]:

(5) $$\mu =1+\frac{2\mu_{0}M_{s}^{2}\sin^{3}\theta }{2\mu_{0}M_{s}H+K\left(3\sin\left(2\varphi -\theta \right)+\sin\left(3\theta -2\varphi \right)\right)},$$

where φ is the angle between the anisotropy axis and the circular direction, and θ is the angle between the magnetization and the circular direction. In this case, *H* and θ are related by the expression *µ*_0_*M_s_H*cos θ = *K*sin (2θ − 2φ).

An analysis of Expression (5) shows that there will be no increasing section on the dependence *µ*(*H*) at φ ≥ 54°. In other words, the increasing section may be absent even in the presence of a rather significant circular component in the anisotropy. Assuming that the anisotropy of the outer layer is predominantly circular, the formula φ = asin [(*r_3_*/*r_2_*)^3^ *M_r_/M_s_*] was used to estimate the average orientation of the anisotropy axes in the inner regions of the wire. We obtained approximately 50°, which is very close to the value of 54° mentioned above.

The greater the number of layers *n* in the wire model, the more detailed the radial distribution of magnetic permeability can be studied. In addition, the contributions of both the frequency dispersion of the magnetic permeability and the domain structure may be taken into account for further research.

The field of the application of the magnetoimpedance tomography method is not limited to MI materials of cylindrical geometry. Further development of the approach will allow the use of magnetoimpedance tomography for the other existing types (amorphous and nanocrystalline ribbons, thin films, and complex 3D magnetic structures) of MI materials [40,41,45,46].

## 4. Conclusions

The article presents a description of the specific implementation of the magnetoimpedance tomography method based on the use of a computer simulation by the finite element method. The application of the method is considered on the example of composite Cu_98_Be_2_/Fe_20_Co_6_Ni_74_ wires of the “highly conductive core–soft magnetic coating” type and amorphous rapidly quenched Co_66_Fe_4_Nb_2.5_Si_12.5_B_15_ wires. The computer model of the wire consisted of *n* coaxial cylindrical regions, each of which was assigned its own value of electrical conductivity and magnetic permeability. A frequency dependence of the impedance with the smallest deviation from the modeled dependences for various combinations of electrical conductivities and magnetic permeabilities of the wire’s regions was found. Thus, we drew conclusions about the real distribution of the electrical conductivity and magnetic permeability over the wire cross-section. Then, the magnetic field dependences of the magnetic permeability of the wire regions were obtained by performing these steps for the impedance experimental dependences obtained in various magnetic fields.

The electrical conductivity distribution over the cross-section of the CuBe/FeCoNi composite wire was obtained as well as the dependence of the magnetic permeability of its coating on the strength of the external magnetic field.

The radial distributions of the magnetic permeability at *n* equal to 2 and 3 and its dependence on the strength of the external magnetic field were determined for the amorphous CoFeNbSiB wire.

In all cases, the results of the magnetoimpedance tomography did not contradict the established ideas about the distribution of the electrical and magnetic properties over the cross-section of the composite and amorphous rapidly quenched wires. Furthermore, our results reveal these distributions in more detail.

## Figures and Tables

**Table 1:** Modeling parameters.

Number of Layers	*n* = 2	*n* = 3		
*r*_1_, μm	76	76		
*r*_2_, μm	90	87	88	89
*r*_3_, μm		90		
Model designation	m76-90	m76-87-90	m76-87-90	m76-89-90

## Data Availability

Data are available from the corresponding author upon reasonable request.

## References

[1] Beach R.S., Berkowitz A.E. (1994). Giant Magnetic Field Dependent Impedance of Amorphous FeCoSiB Wire. Appl. Phys. Lett..

[2] Nakai T. (2020). Sensitivity of Thin Film Magnetoimpedance Sensor in 0.3 T Surface Normal Magnetic Field. IEEJ Trans. Electr. Electron. Eng..

[3] Buznikov N.A., Svalov A.V., Kurlyandskaya G.V. (2021). Influence of the Parameters of Permalloy-Based Multilayer Film Structures on the Sensitivity of Magnetic Impedance Effect. Phys. Met. Metallogr..

[4] Bukreev D.A., Derevyanko M.S., Golubev D.N., Moiseev A.A., Semirov A.V. (2022). The Magnetic Prehystory and Stress-Impedance Effect in Amorphous CoFeNbSiB Wires. Phys. Met. Metallogr..

[5] Popov V.V., Buznikov N.A. (2020). Modeling the Giant Magnetoimpedance Effect in Amorphous Microwires with Induced Magnetic Anisotropy. Phys. Met. Metallogr..

[6] Beach R.S., Smith N., Platt C.L., Jeffers F., Berkowitz A.E. (1996). Magneto-impedance Effect in NiFe Plated Wire. Appl. Phys. Lett..

[7] Tandon P., Mishra A.C. (2022). The Effect of Magnetic Field Orientation on the Magnetoimpedance of Electroplated NiFeCo/Cu Wire. J. Mater. Sci. Mater. Electron..

[8] Wang K., Tajima S., Asano Y., Okuda Y., Hamada N., Cai C., Uchiyama T. (2020). Detection of P300 Brain Waves Using a MagnetoImpedance Sensor. Int. J. Smart Sens. Intell. Syst..

[9] Chen J., Li J., Li Y., Chen Y., Xu L. (2018). Design and Fabrication of a Miniaturized GMI Magnetic Sensor Based on Amorphous Wire by MEMS Technology. Sensors.

[10] Fodil K., Denoual M., Dolabdjian C., Treizebre A., Senez V. (2016). In-Flow Detection of Ultra-Small Magnetic Particles by an Integrated Giant Magnetic Impedance Sensor. Appl. Phys. Lett..

[11] Landau L.D., Lifshitz E.M. (1960). Electrodynamics of Continuous Media.

[12] Masumoto T., Ohnaka I., Inoue A., Hagiwara M. (1981). Production of Pd-Cu-Si Amorphous Wires by Melt Spinning Method Using Rotating Water. Scr. Metall..

[13] Vázquez M., Hernando A. (1996). A Soft Magnetic Wire for Sensor Applications. J. Phys. D Appl. Phys..

[14] Atalay F.E., Kaya H., Atalay S. (2006). Unusual Grain Growth in Electrodeposited CoNiFe/Cu Wires and Their Magnetoimpedance Properties. Mater. Sci. Eng. B.

[15] Atalay F.E., Atalay S. (2005). Giant Magnetoimpedance Effect in NiFe/Cu Plated Wire with Various Plating Thicknesses. J. Alloys Compd..

[16] Usov N.A., Antonov A.S., Lagar’kov A.N., Granovsky A.B. (1999). GMI Spectra of Amorphous Wires with Different Types of Magnetic Anisotropy in the Core and the Shell Regions. J. Magn. Magn. Mater..

[17] Chen D.-X., Pascual L., Fraga E., Vazquez M., Hernando A. (1999). Magnetic and Transport Eddy-Current Anomalies in Cylinders with Core-and-Shell Regions. J. Magn. Magn. Mater..

[18] Buznikov N.A., Antonov A.S., Rakhmanov A.A. (2009). Effect of Torsional Stresses on the Magnetoimpedance of Amorphous Wires with Negative Magnetostriction. Techn. Phys..

[19] Usov N., Antonov A., Granovsky A. (1997). Theory of Giant Magneto-Impedance Effect in Composite Amorphous Wire. J. Magn. Magn. Mater..

[20] Gromov A., Korenivski V. (2000). Electromagnetic Analysis of Layered Magnetic/Conductor Structures. J. Phys. D Appl. Phys..

[21] Kurlyandskaya G.V., Bebenin N.G., Vas’kovsky V.O. (2011). Giant Magnetic Impedance of Wires with a Thin Magnetic Coating. Phys. Met. Metallogr..

[22] Kurlyandskaya G.V., Barandiarán J.M., Gutiérrez J., García D., Vázquez M., Vas’kovskiy V.O. (1999). Magnetoimpedance Effect in CoFeNi Plated Wire with Ac Field Annealing Destabilized Domain Structure. J. Appl. Phys..

[23] Antonov A.S., Buznikov N.A., Prokoshin A.F., Rakhmanov A.L., Iakubov I.T., Yakunin A.M. (2001). Nonlinear Magnetization Reversal in Copper-Permalloy Composite Wires Induced by a High-Frequency Current. Techn. Phys. Lett..

[24] Eggers T., Thiabgoh O., Jiang S.D., Shen H.X., Liu J.S., Sun J.F., Srikanth H., Phan M.H. (2017). Tailoring Circular Magnetic Domain Structure and High Frequency Magneto-Impedance of Melt-Extracted Co 69.25 Fe 4.25 Si 13 B 13.5 Microwires through Nb Doping. AIP Adv..

[25] Shen H., Liu J., Wang H., Xing D., Chen D., Liu Y., Sun J. (2014). Optimization of Mechanical and Giant Magneto-Impedance (GMI) Properties of Melt-Extracted Co-Rich Amorphous Microwires. Phys. Status Solidi A Appl. Mater. Sci..

[26] Sarkar P., Basu Mallick A., Roy R.K., Panda A.K., Mitra A. (2012). Structural and Giant Magneto-Impedance Properties of Cr-Incorporated Co–Fe–Si–B Amorphous Microwires. J. Magn. Magn. Mater..

[27] Knobel M., Sánchez M.L., Gómez-Polo C., Marín P., Vázquez M., Hernando A. (1996). Giant Magneto-impedance Effect in Nanostructured Magnetic Wires. J. Appl. Phys..

[28] Semirov A.V., Gavrilyuk A.A., Kudryavtsev V.O., Moiseev A.A., Bukreev D.A., Semenov A.L., Ushchapovskaya Z.F. (2007). The Effect of Annealing on Impedance Properties of Elastically Deformed Soft Magnetic Wires. Russ. J. Nondestruct. Test..

[29] Alekhina I., Kolesnikova V., Rodionov V., Andreev N., Panina L., Rodionova V., Perov N. (2021). An Indirect Method of Micromagnetic Structure Estimation in Microwires. Nanomaterials.

[30] Volchkov S.O., Pasynkova A.A., Derevyanko M.S., Bukreev D.A., Kozlov N.V., Svalov A.V., Semirov A.V. (2021). Magnetoimpedance of CoFeCrSiB Ribbon-Based Sensitive Element with FeNi Covering: Experiment and Modeling. Sensors.

[31] Kozlov N.V., Chlenova A.A., Volchkov S.O., Kurlyandskaya G.V. (2020). The Study of Magnetic Permeability and Magnetoimpedance: Effect of Ferromagnetic Alloy Characteristics. AIP Conf. Proc..

[32] Yang Z., Chlenova A.A., Golubeva E.V., Volchkov S.O., Guo P., Shcherbinin S.V., Kurlyandskaya G.V. (2019). Magnetoimpedance Effect in the Ribbon-Based Patterned Soft Ferromagnetic Meander-Shaped Elements for Sensor Application. Sensors.

[33] Melnikov G.Y., Lepalovskij V.N., Svalov A.V., Safronov A.P., Kurlyandskaya G.V. (2021). Magnetoimpedance Thin Film Sensor for Detecting of Stray Fields of Magnetic Particles in Blood Vessel. Sensors.

[34] Chiriac H., Barariu F., Neagu M., Vinai F., Ferrara E., Marinescu C.S. (1999). Effect of Mo and Nb Additions on the Magnetic Properties of CoFeSiB Amorphous Wires. J. Non Cryst. Solids.

[35] Bukreev D.A., Derevyanko M.S., Moiseev A.A., Semirov A.V., Savin P.A., Kurlyandskaya G.V. (2020). Magnetoimpedance and Stress-Impedance Effects in Amorphous CoFeSiB Ribbons at Elevated Temperatures. Materials.

[36] Usov N.A., Antonov A.S., Lagar’kov A.N. (1998). Theory of Giant Magneto-Impedance Effect in Amorphous Wires with Different Types of Magnetic Anisotropy. J. Magn. Magn. Mater..

[37] Drexler E.S., Simon N.J., Reed R.P. (1992). Properties of Copper and Copper Alloys at Cryogenic Temperatures.

[38] Hecker M., Tietjen D., Wendrock H., Schneider C.M., Cramer N., Malkinski L., Camley R.E., Celinski Z. (2002). Annealing Effects and Degradation Mechanism of NiFe/Cu GMR Multilayers. J. Magn. Magn. Mater..

[39] Chlenova A.A., Moiseev A.A., Derevyanko M.S., Semirov A.V., Lepalovsky V.N., Kurlyandskaya G.V. (2017). Permalloy-Based Thin Film Structures: Magnetic Properties and the Giant Magnetoimpedance Effect in the Temperature Range Important for Biomedical Applications. Sensors.

[40] Wang T., Zhou Y., Lei C., Luo J., Xie S., Pu H. (2017). Magnetic Impedance Biosensor: A Review. Biosens. Bioelectron..

[41] García-Arribas A., Martínez F., Fernández E., Ozaeta I., Kurlyandskaya G.V., Svalov A.V., Berganzo J., Barandiaran J.M. (2011). GMI Detection of Magnetic-Particle Concentration in Continuous Flow. Sens. Actuators A Phys..

[42] Liu J., Du Z., Jiang S., Shen H., Li Z., Xing D., Ma W., Sun J. (2015). Tailoring Giant Magnetoimpedance Effect of Co-Based Microwires for Optimum Efficiency by Self-Designed Square-Wave Pulse Current Annealing. J. Magn. Magn. Mater..

[43] Melo L.G.C., Ménard D., Ciureanu P., Yelon A. (2002). Influence of Surface Anisotropy on Magnetoimpedance in Wires. J. Appl. Phys..

[44] Tejedor M., Rubio H., Elbaile L., Iglesias R. (1993). Surface Magnetic Anisotropy in Amorphous Alloys. IEEE Trans. Magn..

[45] Nakai T. (2022). A Uniform Magnetic Field Generator Combined with a Thin-Film Magneto-Impedance Sensor Capable of Human Body Scans. Sensors.

[46] Corrêa M.A., Viegas A.D.C., da Silva R.B., de Andrade A.M.H., Sommer R.L. (2007). Magnetoimpedance of Single and Multilayered FeCuNbSiB Films in Frequencies up to 1.8GHz. J. Appl. Phys..

